# The Role of Primary Care Physicians in Curtailing Harmful Social Media Trends

**DOI:** 10.7759/cureus.3271

**Published:** 2018-09-07

**Authors:** Abhishek Gupta, Anurag Dhingra

**Affiliations:** 1 Geriatrics, Center for Addiction and Mental Health/University of Toronto, Toronto, CAN; 2 Family Medicine, Star Medical Center, Mississauga, CAN

**Keywords:** cyberpsychology, internet trends, social media risks, tidepod

## Abstract

Social media platforms, such as YouTube and Instagram, have become the latest medium for communication with a vast potential for influencing society. With their rise, a virtual market now exists where attention in the form of "likes," "views," and "followers" is traded for a monetary and psychological benefit. Amid this trade, physically risky behaviors have arisen to become a new attraction for attention, leading to numerous "trends" that encourage the same risk-taking behavior. Such trends, even those with a positive goal, have simultaneously led to injuries and fatalities, which highlights the necessity of a proactive approach to curtail the same. While media outlets and some non-governmental organizations usually highlight the risks of participating in these trends, the healthcare community has yet to have a collective and organized response to extreme social media participation. As such, a collaborative effort involving multiple tiers of the healthcare community is required to successfully prevent vulnerable populations from falling prey to the virtual attention-based economy of extreme social media participation.

## Editorial

The advent of social media has opened new avenues for access to information and peer communication. While these avenues have had numerous positive effects, they have also given rise to disturbing new trends, some with significant physical and mental harm as a consequence. Recently, emergency rooms across the world have received injured adolescents with curious etiologies often motivated by social media trends that encourage risky behavior. To compound the situation, the certain physical harm from such behavior is not treated as a deterrent but rather as a motivating factor to further one's standing among the vast Internet-based audiences that constitute a virtual attention-based economy.

A prime example is the trend of eating laundry detergent capsules, colloquially known as the "TidePod Challenge." Single-use liquid detergent capsules (SUDS) encased in a water-soluble (polyvinyl alcohol) membrane are mechanically strong and are designed for quick release at the slightest contact with moisture. This can include moist hands or the salivary surface of the human oral cavity. Due to their similar appearance to candy-like products, these capsules are often orally ingested by children, primarily under five years of age. However, this can be attributed to the developmental stage of this demographic, where an oral exploration of surroundings is common [1]. Among the adolescent and older populations, the American Association of Poison Control Centres (AAPCC) reported 39 and 53 cases of intentional exposures (in the years 2016 and 2017, respectively). Within the first 15 days of 2018, AAPCC reported 39 such cases among the age group of 13-19, where 91% were deliberate oral ingestions, coinciding with a rise in Internet-based videos showing intentional consumption of SUDS [2]. This indicates that an alternative underlying incentive drives this adolescent behavior (i.e., harmful ingestions) despite the developed mental capacity and experience to anticipate the harmful consequences. While adolescents could recognize that eating these SUDS would be harmful, videotaping and advertising this consumption on social media created an attraction. This attraction translated into “views,” fulfilling the psychological desire in the risk-taker for greater attention.

Similarly, the “salt and ice challenge” has been a popular trend among adolescents, most common among those aged 12. The activity involves participants applying salt followed by ice to a local body surface. The resultant endothermic reaction creates localized temperatures below freezing point, leading to a burning sensation with thermal injuries consistent with second-degree burns. The resulting injury is very similar in gross appearance and histopathology to multiple bullous diseases as well. Popularized on social media platforms, Roussel et al. [3] discovered 167,000 videos of this phenomenon on various platforms, with some having been seen 36,420,000 times. With such vast amounts of attention available as a reward, there is a clear and significant incentive to engage in risks despite the evident physical danger.

Other "trends" on social media have also developed over recent years and have received varying degrees of media attention, each with unique significant physical risks. While media attention has often led to a mass awareness of such online trends, it has also led to hyperbolic reactions that equate sporadic incidents to widespread epidemics. For example, the “Condom Challenge,” which involved severe aspiration risks from purposely inhaled physical contraceptives, was popularized by the media as an alarming epidemic. In reality, despite the numerous number of videos related to the "challenge" on YouTube, the vast majority were of individuals discouraging such behavior while a very rare number of actual participation incidents were confirmed [4]. This is a significant indicator of another aspect of social media trends, relating to the medium of their public awareness and the hyperbolic exaggeration that often accompanies them. To date, predominantly conventional media outlets have made the public aware of rising social media trends as a potential danger. In the absence of an official body that may monitor social media for rising patterns of public health risks, there is also a distinct lack of concrete data on how extensive participation is in a particular trend. As such, conventional media outlets, while being the sole alarm for dangerous epidemics, can potentially misrepresent the scale of a trend’s participation, exaggerating it in order to promote its danger.

The general underlying principle behind the above “trends” is the use of social media to advertise oneself to a vast audience on the virtual spectrum. Activities that owe their fame to risky behavior invite greater participation in exchange for "likes," "retweets," and "views," a modern form of currency within the attention-based economy of popular social platforms such as YouTube and Twitter. Even dangerous variations of otherwise harmless activities are developed to harvest this modern virtual currency. Thus, participants indulge in such behaviors towards the ultimate goal of greater social acceptance and higher social standing among adolescent groups, often at risk of developing severe bodily harm.

In light of such developments, it is epidemiologically necessary to recognize deficiencies in the public health policy concerning the adolescent individuals’ use of social media. Current behavioral screens for adolescents attending elementary and secondary education include three questionnaires, namely, the Youth Risk Behavior Surveillance System (YRBS), School Health Policies and Practices Study (SHPPS), and School Health Profiles (SHP). These screening programs incorporate various recognized risk factors such as sexual behavior, vaccinations, eating habits, and so on. However, they lack any concrete parameters for the monitoring and guidance of youth regarding the usage of social media. Further, these programs monitor unintentional injuries only, largely ignoring intentionally committed injuries, which are performed for the sake of public/social media attention, altogether [5].

To correct these deficiencies, minor adjustments can be implemented within the epidemiological framework already in place, with potentially significant benefits. Since the core issue is that the desire for attention and psychological approval overrides an individual’s sense of risk avoidance and ideal thought process, the adjustments must reinforce the latter values. Advising adolescents and other at-risk demographics to practice a risk analysis before committing to any actions or following ideation suggested by Internet-based sources is the ideal path. This can take place during elementary and secondary school-based behavior surveys and educations programs, which already address risky Internet use to some degree. Further, the Center for Disease Control (CDC) and poison control centers regularly track rising trends in particular forms of emergency room patient visits. Such databases can be instructed to also monitor patterns in intentional injuries, especially those motivated by social media. This would ideally serve as a warning of impending epidemics, a critical step in proactively discouraging the same risky behavior via public health campaigns. Lastly, community leaders, including public government officials and physicians, can avoid engaging in social media trends as part of community relations in order to avoid the endorsement of risky behaviors. At the very least, public health organizations can issue health risks and recommendations for "trending" social media phenomenon with potential for physical injury.

In conclusion, the healthcare community must recognize social media’s vast, virtual attention-based economy as a significant risk for the psychologically vulnerable youth of today. Based on a virtual economy that rewards risks with public attention and, subsequently, public and peer approval, “trends” have immense potential for physical and psychological abuse. Participants attempt physically risky maneuvers on video recordings to advertise their ingenuity and bravado, which is rewarded by the increased Internet-based attention that translates into psychological approval. To combat this risk, school-based risk screenings and public health campaigns should incorporate education against social media risks. Youth must be advised against the following Internet-based trends for the sake of public approval or without performing due diligence via a risk analysis. Public health organizations should also incorporate exotic intentional injuries in their epidemiological surveillance programs in order to identify upcoming trends in social media with physical risks. The healthcare community has a long tradition of adapting to new technologies and the risks that accompany them. Whether it is in the form of sexual counselors at secondary schools or seat-belt usage advisories, health risk management is a collaborative effort requiring investment from multiple social health entities. Therefore, social media is a risk whose appropriate use and risks must be taught to increasingly technologically involved generations via a collaborative effort at all levels of society.
